# Community mitigation decisions in elephant conflict zones of southern India depend on environmental and socio-economic drivers

**DOI:** 10.1038/s41598-025-14867-3

**Published:** 2025-10-06

**Authors:** Simran Prasad, Vikram Aditya, Jennifer Solomon, Krithi K. Karanth

**Affiliations:** 1 Centre for Doctoral Studies, Manipal Academy of Higher Education, Manipal, 576104 Karnataka India; 2 Centre for Wildlife Studies, 37/5, Yellappa Chetty Layout, Bengaluru, 560042 Karnataka India; 3 Department of Human Dimensions of Natural Resources, Colorado State University, Fort Collins, CO USA; 4 Nicholas School of the Environment, Duke University, Durham, NC USA

**Keywords:** Human–wildlife interactions, Human–wildlife conflict, Megafauna, Mitigation techniques, Conservation, Community perspectives, Conservation biology, Environmental social sciences, Environmental impact

## Abstract

Human–elephant conflict is a major threat to people and elephants across Asia and Africa. Understanding the factors influencing people’s decision to implement mitigation measures is crucial to devise better conflict mitigation measures. We surveyed 507 rural communities in elephant dominated landscapes, across four districts in southern India using snowball and opportunistic sampling. Fourteen covariates were analysed through a mixed-methods approach, using Classification and Regression Trees (CART) for quantitative analysis, and thematic analysis for qualitative insights. Three key drivers: rainfall, land ownership and proximity to water bodies shaped mitigation decisions. The CART revealed two distinct decision paths. In Path 1, households with lower rainfall and smaller landholdings had a 68% likelihood of adopting mitigation measures. In Path 2, households experiencing higher rainfall and bigger landholdings, closer to water bodies, had a 7% likelihood of adopting mitigation measures. Notably, trenches were linked to elephant injuries, while solar and electric fencing were associated with elephant deaths, indicating poor design and maintenance of these mitigation measures resulting in elephant casualties. Despite the conflict, communities expressed sadness over elephant casualties, reflecting strong cultural ties. Our findings underscore the necessity for proactive measures, including early warning systems, patrolling networks and regenerative agricultural practices to promote biodiversity. As global interest in conflict mitigation grows, integrating local knowledge is essential for community-based management in shared landscapes.

**Supplementary Information:**

The online version contains supplementary material available at 10.1038/s41598-025-14867-3.

## Introduction

In many parts of the world, humans and wildlife have coexisted for centuries^1,2^. Human–wildlife interactions are increasingly common in shared landscapes particularly in densely populated countries in Asia^3,4^. These interactions can sometimes result in negative, hostile interactions, termed as human–wildlife conflict that can endanger people and wildlife^3,5^. Conflict can have serious consequences, placing threatened wildlife species at risk and causing significant damages to people, i.e. crop and property loss, livestock depredation, injury and even death to humans^6,7^ .

Across the world, conflict with elephants occurs frequently, leading to widespread impacts and casualties^7,8^. Elephants are the world’s largest megaherbivores, and make a significant impact on tropical forest vegetation and surrounding habitat through their foraging activity and extensive movement^9^. Due to their large body sizes, wild Asian elephants (*Elephas maximus)* often spend 12–18 h per day feeding, and can consume up to 10% of their body mass in fodder^9–12^. Elephants also facilitate forest regeneration and maintain ecological integrity by dispersing seeds away from parent trees^13^. Further, the paths created as they move through dense vegetation helps the movement of small mammals, and the forest gaps can provide vegetation for other herbivores to forage^11^. However, the ecological requirements (food and water), and specific traits (large home ranges and seasonal migration patterns) predispose elephants to conflict when habitats shrink and forests are fragmented^9,11,12,14^. Increased habitat fragmentation has resulted in elephants facing ecological trade-offs, where the need for critical resources like food and water outweighs the risk of entering human-dominated areas^15^.

Consequently, human–elephant conflict has emerged as a significant conservation challenge throughout the elephant range, often resulting in substantial repercussions to both people and elephants^3,7,8^. As Sukumar (2003) indicates, the origins of human–elephant conflict can be traced back to over 10,000 years ago, with the emergence of agriculture in the Old World when cultivation of palatable crops to elephants, such as paddy (*Oryza sativa)*, cereal, and vegetables (potato, carrot, pumpkin, etc.) increased human–elephant encounters^9^. In India, the world’s most populous country with approximately 473 people per km²^16^, elephants are often in conflict with humans, with 500 people and 100 elephants killed annually due to conflict^7^. Previous research has indicated that approximately 500,000 families have faced direct effects of crop damage in India annually, highlighting the severity of this conflict^17^. Driven by escalating conflict, anthropogenic impacts and habitat degradation, wild Asian elephant populations in India face formidable threats to their survival^7,8,12,14^.

For decades, elephant movement in these landscapes has been driven by environmental factors (i.e. habitat fragmentation, crop and water availability), shaping foraging needs, especially during dry seasons^18^. Previous research by Sukumar et al. (1989) highlights these patterns across tropical forests in southern India, with elephants utilizing habitat based on seasonality, precipitation patterns and consumption of palatable crops^19^. According to Anoop et al. (2023), these environmental patterns are still prevalent in the landscape, with habitat use of elephants highly dependent on water sources with greater forest cover, particularly in areas with lower human disturbance^20^. This trend is also observed across continents, with research highlighting that African elephants (*Loxodonta africana)* in Kenya spend less time in fragmented landscapes due to low forage potential^21^.

In addition to environmental drivers, elephant behaviour can also impact movement patterns, often occurring in proximity to populated areas^7,8,12,14^. Due to increasing anthropogenic pressures in their landscapes, elephants have modified their behaviour, changing their time-activity budget as a behavioral response to their evolving environment^22^. Oftentimes, these modifications can result in long-term changes in home ranges and movement patterns, especially due to land-use transformations and global climatic shifts^22^. Research by Srinivasaiah et al. (2019) on male Asian elephants in India found that behaviour adaptations, such as forming all-male groups in fragmented habitats lead to their survival in urbanised landscapes^23^.

Due to drivers of conflict being both ultimate and proximate, managing conflict can be difficult for people living near wildlife. Ultimate factors are rooted in evolutionary traits of elephants, including long-distance migration patterns, consuming large amounts of fodder and water daily, and utilizing extensive home ranges for resource requirements^9,11,19^. These traits, which evolved over millenia in relatively undisturbed habitats, now predispose elephants to conflict in increasingly human-dominated landscapes^1,9^. In contrast, proximate causes are immediate, context-specific triggers of conflict, such as poor functioning or inoperable mitigation measures, inefficient crop guarding activities and cultivation of crops within elephant ranges^9,12,14^. Together, these drivers escalate conflict patterns and create significant challenges for communities, particularly for forest dependent people already struggling with resource scarcity^1,24^. These dynamics become even more intricate when considering behavioural adaptations of elephants, including shifts in movement patterns and social structures, in response to habitat fragmentation and anthropogenic pressures^22^.

As a result of conflict, people often deploy mitigation measures to reduce encounters, including passive measures such as electric fencing and physical barriers that enable active guarding by farmers^25^, or active measures such as translocation, acoustic and visual deterrents (e.g., firecrackers and flaming torches, local drives and even captures for keeping elephants in captivity)^6,26^. If mitigation measures are poorly designed or maintained, such as non-functional barriers, this can intensify conflict and lead to communities facing severe damages^11,12,14^. Further, strategies such as active guarding deprives farmers of sleep and compromises health and psychological welfare in the long term^24^.

When evaluating the effectiveness of various passive and active mitigation measures, it is apparent that a combination of measures, within particular environmental and cultural contexts can comprehensively tackle the nuances of human–wildlife conflict, especially for positive results in the long-term^25^. Studies have focused on the benefits of passive measures (i.e., physical barriers and electrical fencing) as these methods are cost-effective and robust in diverting elephants^26,27^, with the use of electric fencing in Bhutan reducing crop loss for communities significantly, and minimizing crop guarding time and effort^28^. Despite these benefits, passive measures can also prove ineffective in certain landscapes and conflict situations. For instance, barriers such as electric fences can prevent elephants from entering human-occupied areas, but their success depends on a robust design, regular maintenance and ensuring an adequate pulse voltage. Sukumar (2003) and Lenin & Sukumar (2011) emphasize that without these considerations, such fences are prone to failure^9,12^. Other barriers, including elephant proof trenches spanning several kilometres have also been deployed in Asia and Africa^7,9,12^. Although these can work well when combined with electric fences, they can be ineffective if poorly maintained^12^. Rubble walls are more effective when deployed with electric fences, but are often costly to construct and maintain over time^12^.

Active measures (i.e., acoustic and visual deterrents) have demonstrated positive responses in southern India, with sound-playback systems of leopard (*Panthera pardus)* and tiger (*Panthera tigris)* growls, significantly deterring elephants from crop raiding^29^. However, certain visual deterrents, such as firecrackers can be dangerous to people at times, accidentally wounding them during a conflict encounter^30^. Therefore, identifying a combination of landscape-specific, mitigation measures that work in tandem during wildlife interactions is key^25^. In contrast, mitigation measures can also negatively impact elephants, with poorly laid fences connected to the main transmission lines instead of a regulated source of power supply, causing injuries and even electrocution^3,25,31,32^.

As people living in these areas often encounter elephants and face interactions, these experiences, coupled with religious beliefs and socio-demographic drivers (i.e., age, education levels) can fundamentally shape perception of elephants leading to behaviors influenced in part from past experiences^33,34^. Perspectives on elephants can fluctuate, often resulting in lower tolerance and retaliatory killings when there are frequent conflict encounters and increased frustration towards elephants^34^. Due to escalating, widespread conflict, it is crucial to understand socio-demographic and environmental drivers that shape people’s ability to implement mitigation measures, and their perceptions of elephants and conservation measures. This knowledge can be utilized to develop solutions that reduce conflict and conserve elephant populations globally, especially in conflict-prone landscapes with vulnerable communities of people^33,34^. Further, local communities often have experiential knowledge, gained from their lived experiences with elephants^3,6,15,32^. These experiences provide unique insights into species population trends, movement patterns and human–wildlife interactions^31^.

We investigated the key drivers influencing community-decision making for mitigation measure deployment of elephant conflict in the Western Ghats, a global biodiversity hotspot and a key elephant conservation landscape in India. The Western Ghats is particularly relevant and environmentally significant for elephant populations, with a reported 25% of the global population of Asian elephants present within this landscape^35^. While other studies have extensively documented the impacts of human-elephant conflict, our research uniquely focuses on the decisions influencing mitigation measure deployment, and thereby addresses a critical gap in understanding how socio-demographic, environmental, and experiential factors shape conflict management. Recent studies in the Western Ghats have reported an increase in human casualties, predominantly linked to crop and property damage cases, driven by habitat degradation due to anthropogenic pressures^36^. Similarly, another study in North Bengal revealed how cultural, economic and social drivers can shape community attitudes toward conflict management^37^. However, these primarily focus on the consequences of conflict, rather than a direct examination of the decision process for deploying mitigation measures.

By focusing on the drivers of mitigation measure deployment and the wealth of community knowledge, our study fills a critical gap. We provide evidence-based insights that can guide adaptive elephant conservation and management measures across shared landscapes globally, and can be applicable to people and management within conflict-prone landscapes that are fragmented and home to a dense human population, such as the Western Ghats. Our objectives were to:

1. Assess key socio-demographic (age, education levels, etc.), economic (financial status) and environmental drivers (land cover type, distance to water bodies, etc.) influencing deployment of mitigation measures within the study area.
2. Identify mitigation measures that can potentially compromise elephant survival.
3. Examine perspectives of local community members regarding elephant casualties using thematic analysis to capture experiential information based on people’s lived experiences with elephants.
4. Document and analyse community-suggested mitigation measures for addressing human–elephant conflict within the study area.

## Methods

### Study area

**Fig. 1:**
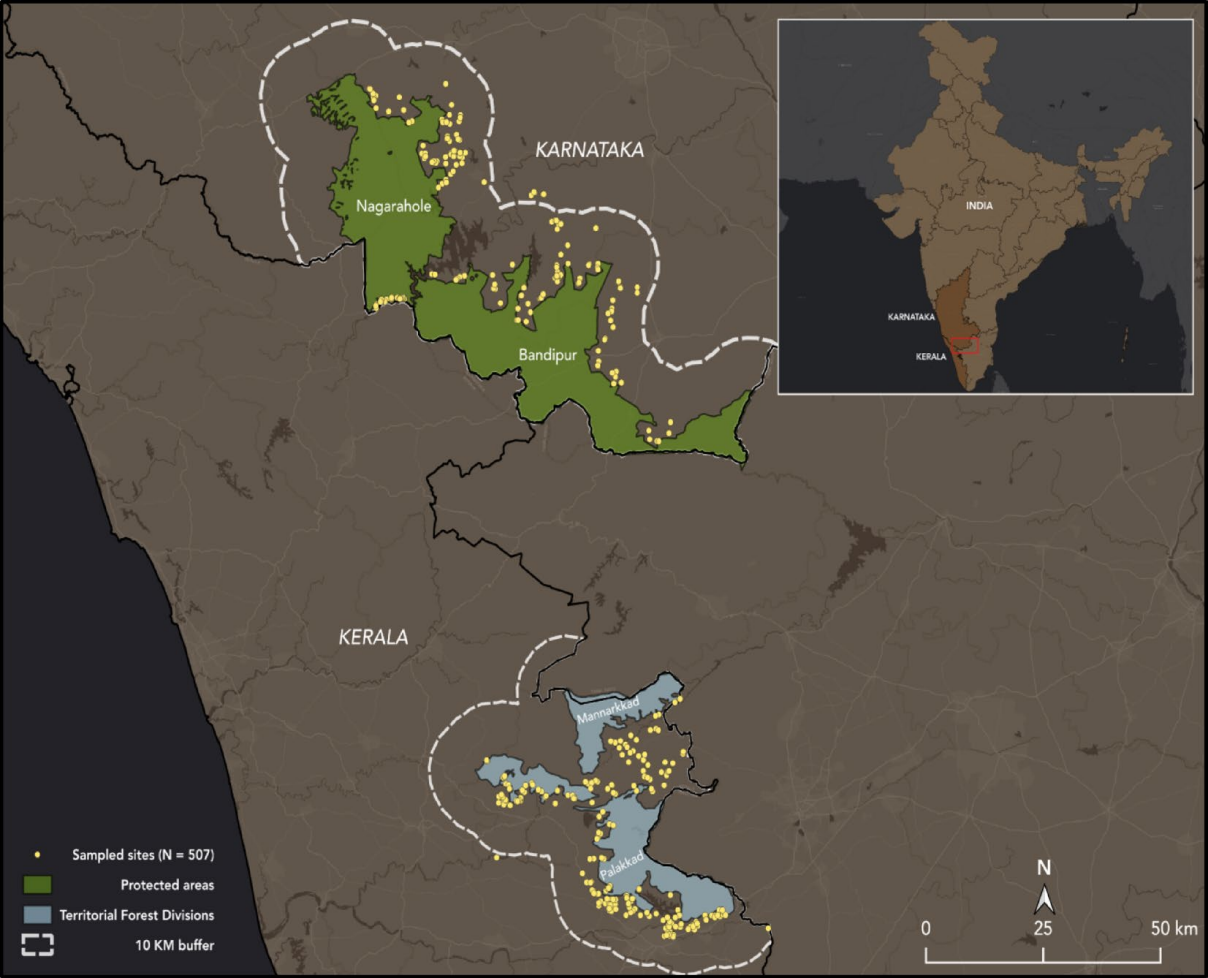
Map showing survey locations in 10 km buffers around PAs across the States of Karnataka and Kerala, India. The State layer and Protected Area boundaries were extracted from the World Database of Protected Areas (WDPA Esri., 2024).

Our study was conducted across four areas in the southern Nilgiris Biosphere Reserve (Fig. 1) in the Western Ghats, in the States of Karnataka and Kerala, India. The four areas were Bandipur and Nagarahole Tiger Reserves in Karnataka, and Palakkad and Mannarkkad Territorial Forest Divisions in Kerala. The Western Ghats is a global biodiversity hotspot hosting several species of endemic fauna and flora^38^. Comprising less than 6% of India’s landmass and covering an estimated area of 160,000 km², spread over six Indian states, the Western Ghats contains more than 30% of all plant and vertebrate species in India^39,40^. Rainfall in the surveyed areas varied between 1000 and 3000 mm, with 1000–1666 mm indicating low rainfall, 1667–2333 indicating moderate rainfall and > 2334 mm indicating high rainfall. Previous research has indicated that the mean annual rainfall varies between 4000 and 7800 mm, and mean daily temperatures can vary from < 5 degrees celsius in the winter, to nearly 40 degrees celsius in the summer, especially across mountainous regions of the Western Ghats^41–43^.

In the State of Karnataka, we conducted our study around Bandipur (~ 900 km^2^) and Nagarahole (~ 640 km^2^) Tiger Reserves^44,45^. These protected areas are home to a diverse assemblage of mammals, such as elephants, tigers, Indian Bison (*Bos gaurus)*, Asiatic wild dog (*Cuon alpinus)*. According to Madhusudan et al., 2015, elephant presence in Karnataka was confirmed in 972 out of Karnataka’s 2855 forest beat divisions, indicating elephant distribution over an area of 38,313 km^2^^46^. In a separate study focusing on habitat occupancy and elephant population density, Jathanna et al., 2015 reported that elephants occupied 13,483 km^2^, approximately 64% of the total available elephant habitat of 21,167 km^2^ in specific regions of the Western Ghats, and elephant population densities within these areas were estimated at approximately two individuals/km^2^^18^. Human population densities in these areas range between 140 and 346 people/km2^40^. The local people in these areas rely on farming practices (i.e., agriculture, dairy farming) and wage labor as a part of their income^48^. These communities have reported recurring crop and property damage, livestock depredation, human injury and death cases due to elephant conflict^47,48^. Communities also regularly farm for subsistence and commercial farming purposes, often growing rice, maize, finger millet, etc^49^. Local people also utilize mitigation techniques on their farmlands (i.e., crop-guarding platforms, wire fencing, electric fence etc.) to prevent conflict damages. Mitigation techniques deployed by authorities include electric fences, trenches, and railway fences^6,25,49,50^.

Our study sites in the State of Kerala were located in the forest of Palakkad (~ 420 km^2^) and Mannarkkad Territorial Forest Divisions (~ 245 km^2^). After reviewing Forest Department records, we identified that these areas have the most crop damages and conflict events reported in the landscape. Both forest divisions host multiple charismatic species, such as elephants, tigers, and endemic species, like the lion-tailed macaque (*Macaca silenus)*^51^. Previous work has indicated that total elephant distribution in southern India covers ~ 39,500 km^2^, encompassing north and south areas of the Palghat Gap, alongside the Eastern and Western Ghats mountain ranges^52,53^. Elephant population densities within the study sites in Kerala were estimated at roughly 1 individual/km²^51,54^. Human population densities range between 318 and 627 people/km²^55,56^. Communities rely on agriculture for subsistence and commercial farming (often growing banana, rice, coconut, etc.), and animal husbandry processes for their income^57,58^, as well as utilizing different mitigation techniques (electric fencing, solar fencing, etc.) to avoid conflict with wildlife^57,58^.

### Data collection

The study was conducted over a seven-month period in 2022. We obtained human subjects IRB ethics approval from the Centre for Wildlife Studies (CWS_IRB_2022_02), and the University of British Columbia (H22-01114). The human research ethics guidelines were followed in accordance with the Tri-Council Policy Statement: Ethical Conduct for Research Involving Humans (TCPS 2), as established by the Panel on Research Ethics, Government of Canada. Participants were over 21 years of age, and we received verbal and or/written consent based on participants’ preference. Our team for the questionnaire survey included the project leader (Principal Investigator) and field assistants. The team were trained to conduct the questionnaires in Kannada and Malayalam (the State languages of Karnataka and Kerala, respectively). To account for socio-cultural differences between the two landscapes, we tailored our survey design accordingly. Our questionnaire was developed as a single survey instrument and adapted to incorporate local terms, practices and beliefs. To ensure linguistic accuracy, the questionnaire was translated to the local language and then back-translated into English by an independent translator that had not viewed the original. The back-translation process allowed us to identify and correct discrepancies, ensuring that the final, local language version maintained the original meaning and intent. Our surveyors were trained to approach questions with sensitivity to the cultural nuances within each landscape. The adaptations were informed through discussions with local community members and Forest Department staff. Training was conducted through role-playing exercises, where team members assumed the roles of different stakeholders. This helped them become familiar with the questionnaire and develop skills to effectively and sensitively engage with diverse participants. We also conducted 40 pilot surveys across the study area to test and refine the questionnaire format and structure, prior to data collection. Our sampling protocol involved utilizing a conditional population of people who experienced direct conflict with elephants, to gather information on socio-demographic variation and perspectives. We used a combination of techniques to identify specific areas of reported elephant conflict. Firstly, we gathered information on past conflict events across our study sites in Karnataka using the Indian Right to Information Act of 2005^59^.

In Kerala, we met with Forest Department staff across Mannarkkad and Palakkad districts, and compiled a list of villages that had reported elephant conflict in the last five years. Therefore, our work was focused on relatively high-impact conflict zones across both States. We approached local community members in the villages that were reporting conflict and used a combination of opportunistic and snowball sampling^60^ to identify individuals who had directly experienced elephant conflict or had witnessed interactions between elephants and community members. Each questionnaire consisted of eight sections (comprising structured, open-ended and semi-structured components), focusing on human–elephant interaction. We requested one person per household, aged 21 years old or more to participate, and this was often the household head or income earner of the family, responsible for the family’s financial wellbeing.

For environmental differences, we conducted land-use and land-cover classifications separately for each landscape to accurately capture their environmental characteristics, using Google Earth Engine. Key environmental drivers (i.e., rainfall, vegetation type, elevation) were included as covariates in our CART model to account for variations. Additionally, State was incorporated as a covariate to ensure that differences between the two landscapes were statistically considered.

### Data analysis

To extract information on environmental drivers (i.e., elevation, land-use/land cover), we used Google Earth Engine^61^ and Sentinel-2 data, having a 10 m spatial resolution. We established training points for different land classes (low, medium and dense vegetation, water bodies, human settlements) and used a random forest algorithm for a supervised classification for the images. We also extracted data on elevation using NASA’s Land Processes Dataset^62^ and precipitation patterns using Climate Hazards Group Dataset^63^. To calculate distance from the waterbody and distance from forest, (calculated from the location of elephant interaction), we used the NNJoin tool in QGIS^64^. All data organization and analysis was conducted using R version 4.3.1^65^.

The selected covariates were identified based on previous research documenting their relationship with elephant conflict (Table 1). We scaled nine numerical variables: (1) Age (2) Household size (3) Duration of residence (4) Average annual income (5) Number of elephant conflict events (6) Distance to water bodies (7) Distance to protected areas (8) Rainfall and (9) Elevation to ensure equal influence in the model. A Pearson’s correlation test was used to check the strength of association between numerical covariates^66^. The analysis also consisted of five categorical variables: (1) Education levels (2) Sex (3) Land cover type (4) Occupation (5) Crop types grown.

**Table 1:** Background information, references, and hypothesized effects of covariates used in the analysis, with justification for their inclusion.

Sl no.	Covariate	Background information	Expected directionality of effect
1	Age of respondent	Previous research indicates that older people are more likely to die from wildlife attacks compared to younger people due to being more susceptible to injuries and deaths ^30,67^. However, there is limited evidence to suggest that older individuals are more likely to deploy mitigation measures in response to wildlife attacks	Age is positively correlated with the decision to deploy mitigation measures
2	Household size	Studies have shown that household size can impact perceptions toward wildlife conservation as more members of the household could be involved in crop guarding and other related mitigation activities^67^	Household size is positively correlated with the decision to deploy mitigation measures
3	Residence time near the forests (within the study area)	People living in proximity to wildlife for decades, and even generations, develop exposure to wildlife interactions, shaping their beliefs and perception of wildlife^33,34^. These perceptions can influence decisions around deploying mitigation measures. For instance, some people may choose not to implement them due to the high costs associated with installation and maintenance, particularly when living in proximity to wildlife^68^	Residence time near the forests is positively correlated with the decision to deploy mitigation measures
4	Average annual income	Low-income households face a disproportionate share of conflict losses compared to other groups^25,69^, often requiring them to invest financially in mitigation measures	Average annual income is negatively correlated with the decision to deploy mitigation measures
5	Number of elephant conflict events	Research indicates that people deploy mitigation measures due to incurring more damages as increasing wildlife conflict events occur to households^25^	The number of conflict events is positively correlated with the decision to deploy mitigation measures
6	Distance to water bodies	Elephants frequently visit areas with water bodies due to their high water requirements (190 L daily) and strong resource dependence^9,11,12^	Distance to water bodies is negatively correlated with the decision to deploy mitigation measures
7	Distance to protected areas	Due to food availability outside of protected areas, elephants forage on croplands, often causing crop damages, etc.^14,15,30^	Distance to protected areas is negatively correlated with the decision to deploy mitigation measures
8	Rainfall	Elephants are particularly sensitive to precipitation changes and seasonality, often moving toward fresh vegetation growth after both large and small rainfall occurrences. Their positive response to vegetation driven by rainfall can shape their movement patterns and interaction with people in shared landscapes^9,70^	Rainfall is positively correlated with the decision to deploy mitigation measures
9	Elevation	Along with rainfall, elevation can influence elephant movement, as these factors can influence seasonality and vegetation dynamics. Previous research indicates that elephants frequent lower elevations during periods of high vegetation productivity; however, they tend to move toward higher elevations when vegetation is less abundant^70^	Elevation is negatively correlated with the decision to deploy mitigation measures
10	Education levels	Social aspects such as level of formal education (primary school, secondary school, etc.) can influence income generated in households, which impacts attitudes towards wildlife^71^. Further, limited formal education could hinder the ability of farmers’ to access information, and effectively engage with or implement mitigation measures^72^	Education levels are positively correlated with the decision to deploy mitigation measures
11	Sex	Previous research has observed that risk perception of wildlife, and attitudes toward wildlife interactions can vary based on sex, with women perceiving a greater risk from wildlife interactions compared to men^73^	Women are more likely to deploy mitigation measures compared to men
12	Land cover type	Vegetation dynamics and changes in landscape can be influenced due to other environmental drivers (i.e. rainfall and elevation) and seasonality, impacting elephant movement and habitat use in landscape^9,11,70^	Households living near fragmented areas are more likely to deploy mitigation measures
13	Occupation	The primary occupation of the household head (i.e., livestock management, farming, etc.) can significantly shape the family income, thus influencing decisions to deploy mitigation measures to avoid conflict^74^	Households reliant on farming more than other occupation types are more likely to deploy mitigation measures
14	Crop types grown	Research has indicated that elephants have palatability for certain crops (i.e., banana, rice, etc.) compared to other crops for foraging^9,11,12,19,75^	Households growing crops such as bananas, rice, millets, etc. are more likely to deploy mitigation measures

To test our hypotheses, we used a recursive partitioning technique called Classification and Regression Trees (CART) to create decision trees^76^. We conducted land-use and land-cover classification separately for each landscape to capture their unique environmental characteristics. Key environmental drivers (i.e., rainfall, vegetation type, elevation) were included as covariates in our CART model. The State was incorporated as a covariate to ensure that differences between the two landscapes were statistically considered. The CART approach can handle nonlinear relationships, and create a decision tree using a supervised learning approach^76,77^. The tree structure is created from the root node, which has all the training data (70% of the total dataset), and undergoes binary recursive partitioning, presenting each step of the decision tree paths until the criterion is met^76,77^. Our input variables were all socio-demographic and environmental drivers (Table 1). The model then created an output of the decision tree that presented the decision paths leading to deployment of mitigation measures or non-deployment of measures. The “caret” and “r-part” packages in R were used to model and visualize the CART. A complexity control parameter was used to prune the tree, and we identified the optimal parameter that is suitable for a robust model structure^76,77^. We also calculated model accuracy, by comparing the number of predictions to the total number of observations within the analysis. Finally, we extracted the variable importance scores, which quantifies the relative contribution of each predictor variable to the overall decision tree and model predictions.

Our analysis also focused on exploring the relationship between reported elephant injuries and deaths due to mitigation measures. Due to smaller sample sizes per category, we used Fisher’s Exact Test^78^ to estimate the significance between both categories (elephant injury vs. elephant deaths).

For open-ended responses, we used Taguette^79^ to code participant responses into themes. Thematic analysis analyzes patterns and themes in data and extracts meaningful interpretations and recurring themes from the data^80^. We analyzed participant answers on mitigation measures (i.e. measures they have deployed, recommendations they have regarding mitigation measures in the region). We coded all responses into themes and sub-themes using inductive coding, with no prior impressions of the data^80^. Following this, we revisited our code-book and transcripts to ensure consistency in coding and identification of broader patterns within the transcripts.

## Results

### Participant characteristics

The survey lasted 40–60 min per respondent and our conditional population (people facing direct conflict with elephants) included 253 participants in Karnataka and 254 participants in Kerala. Our analysis (Sect. 3.3–3.5*)* focused on 470 participants (93% response rate), as thirty-seven respondents declined to participate. Most respondents completed primary schooling (43%), and age categories ranged from 21 to 35 (18.9%), 36–50 (32.3%), 51–65 (33.4%), and over 65 years old (15.3%). 24% (24%) of participants identified as women, and 75% as men. 90% (90%) of participants in Karnataka, and 50% of participants in Kerala had deployed mitigation measures (Supplementary Table 1).

### Exploratory data analysis and model accuracy for land-use classification

Pearson’s correlation values ranged from − 0.75 to 0.37 suggesting strong to moderate correlations between covariates (Supplementary Fig. 1). A Principal Component Analysis (PCA) was done to check the association of predictor and response variables using variance of the data, which indicated 28.9% and 17.5% of variance respectively, and the covariates within the first component was retained for analysis (Supplementary Fig. 2). For the land-use classification models, the training and validation accuracy for the Karnataka data classification model was 95.9% and 94.9% respectively. For the Kerala model, the training and validation accuracy was 96.7% and 97.3% respectively.

### Decision tree path for drivers influencing the decision to deploy mitigation measures

**Fig. 2:**
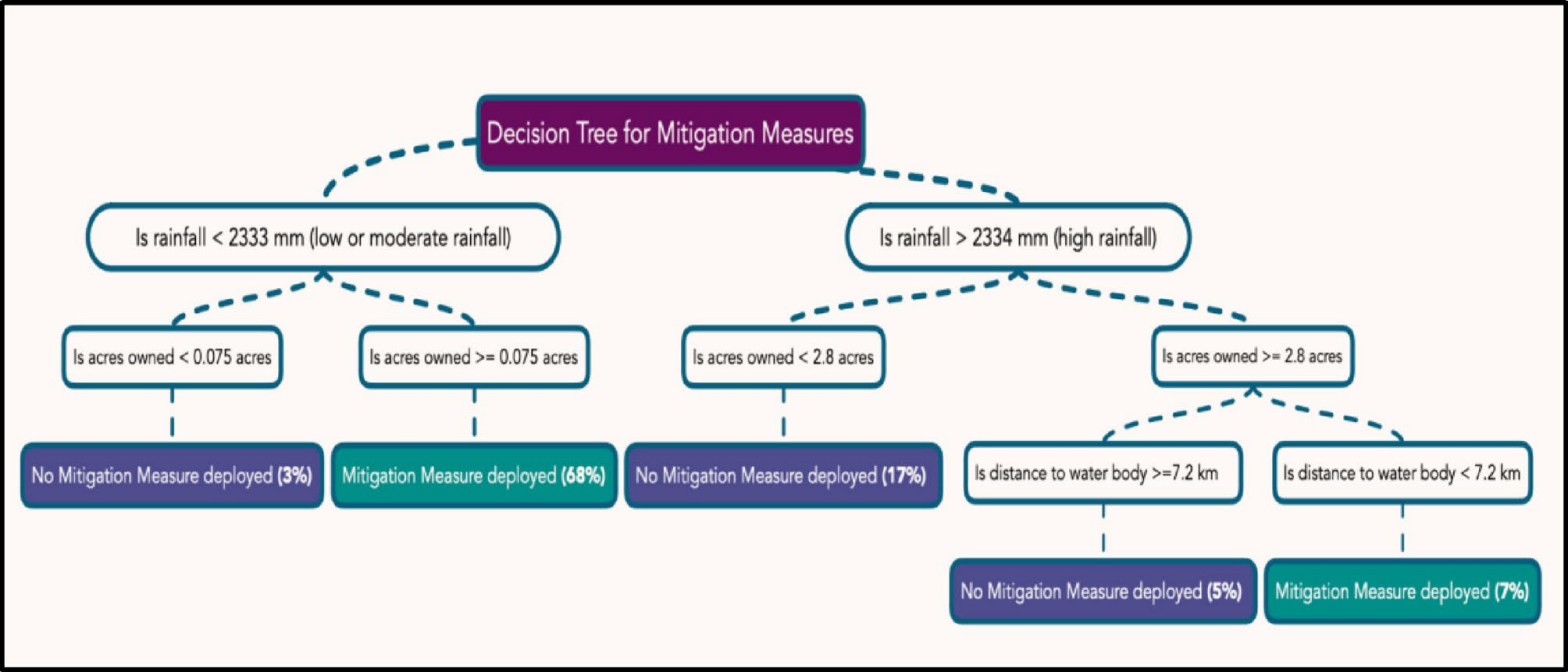
The primary node of the CART model is (1) rainfall, followed by (2) acreage ownership, indicating landholding size, partitioning at 0.075 acres and 2.8 acres. The final, smaller node (3) is the distance of the water body to the location of the elephant interaction and partitions at 7.2 km. There are a total of 5 decision paths that are present. For example, in a situation of low or moderate rainfall, and acres owned more than 0.075 acres, the likelihood of people installing mitigation measures is 68%.

The optimal complexity control parameter was 0.05, and the model accuracy of the CART model was 79.4%. The decision to deploy mitigation measures is primarily influenced by three drivers: rainfall, acreage ownership and distance to water bodies (Fig. 2). Based on our training data, the decision tree reveals that households in areas experiencing low and moderate rainfall (1000–2333 mm) and owning at least 0.075 acres of land are 68% more likely to adopt mitigation measures (Path 1). This pattern indicates that in relatively drier areas with moderate landholdings, there is greater motivation or necessity to actively invest in mitigation measures.

In contrast, in areas with high rainfall (exceeding 2334 mm) and with larger acreage ownership (≥ 2.8 acres), the likelihood of deploying mitigation measures reduces. For example, among those living close to water bodies (within 7.2 km), only 7% are likely to install mitigation measures (Path 2). Proximity to water may be creating challenges, or complicating mitigation deployment.

### Decision tree path for drivers influencing the decision to not deploy mitigation measures

The decision tree analysis reveals distinct decision paths influencing households’ decisions to not adopt mitigation measures (Fig. 2). Households in areas receiving low and moderate rainfall and owning less than 0.075 acres of land are 3% more likely to avoid deploying mitigation measures. This suggests that in drier areas with smaller landholdings, motivation to invest in mitigation is lower. When rainfall was higher, households owning larger land parcels (≥ 2.8 acres) and located farther from water bodies (more than 7.2 km) also tended to avoid mitigation measures. The highest likelihood of not deploying mitigation (17%) is observed in households experiencing high rainfall, but with smaller landholdings of less than 2.8 acres. These paths highlight how relationships between environmental drivers and land ownership influence mitigation decisions, suggesting a potential trade-off that affects people’s choice to invest in such measures.

### Variable importance in CART model

The CART algorithm uses binary recursive partitioning to construct decision trees, which also provides the relative importance of each variable in predicting the outcome. Variable importance in a CART model reflects the total contribution of each variable in reducing classification error across all splits within the decision tree, rather than the first, most significant split. Additionally, pruning the tree using the complexity parameter does remove some splits, which can affect the final variable importance scores.

Our analysis identified 13 variables with importance percentages of the overall CART model, ranging from 0.6 to 28%. Top variables that are important and influence the overall CART model are rainfall (28.0%), elevation (16.8%), acres owned (15.7%), crop type – rubber (8.2%), State (7.4%), crop type – coconut (6.0%), crop type – arecanut (5.5%), distance to water body (4.8%), vegetation type – dense (2.7%), vegetation type – medium (2.1%), number of conflict events (1.3%), crop type – pepper (1.0%) and age (0.6%). These results suggest that environmental factors such as rainfall, and other drivers such as landholding size are crucial in shaping people’s decisions to implement mitigation measures across the landscape.

### Reported elephant injury and death patterns due to mitigation measures across Karnataka and Kerala

Our results indicate statistically significant associations for elephant injury patterns (*p* = 0.004, 95% confidence interval: 0.000–0.573, *n* = 507) in Karnataka compared to Kerala, however no statistically significant associations for elephant death patterns across both States (*p* = 0.5, 95% confidence interval: 0.434–1.569, *n* = 507).

**Fig. 3:**
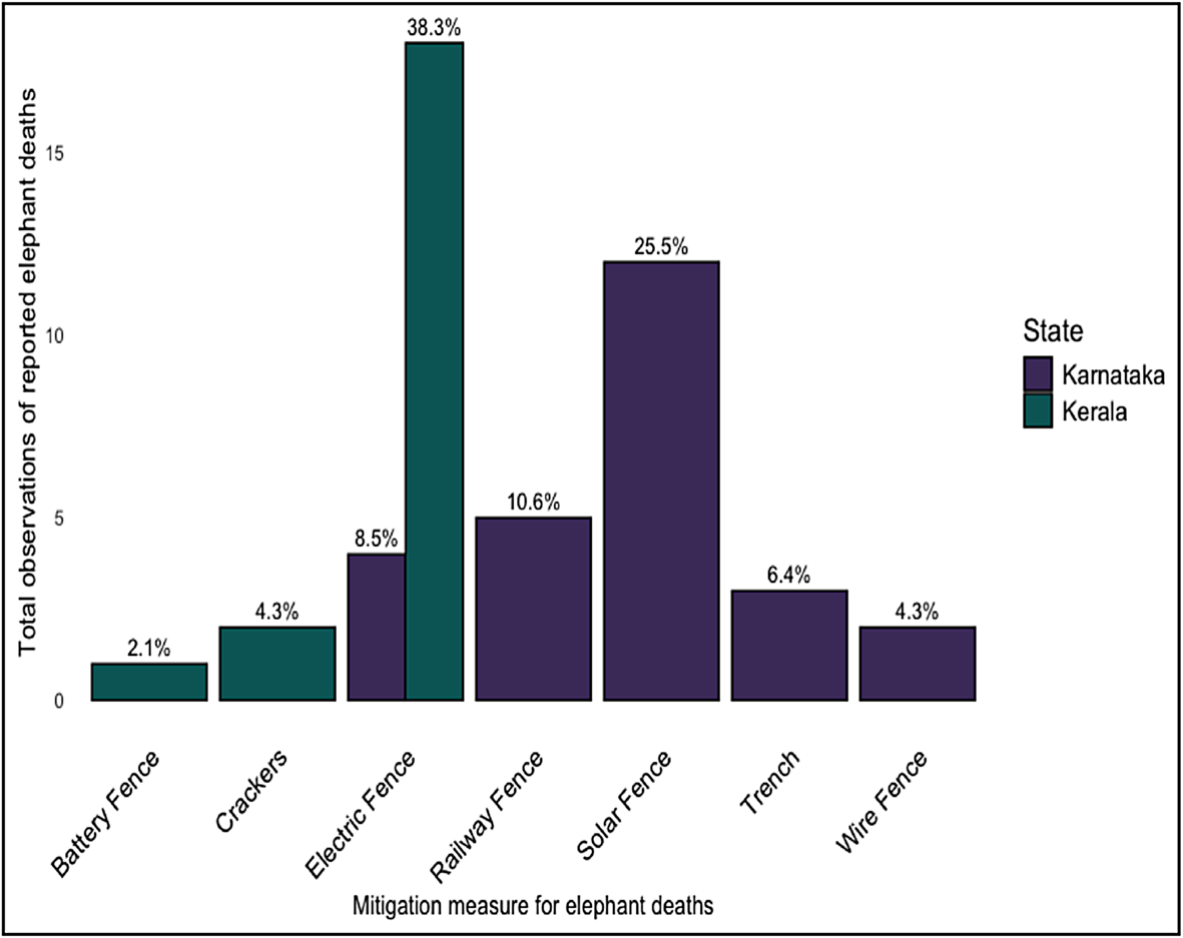
Bar chart of various mitigation techniques impacting elephant deaths. The data on elephant injuries and deaths have been gathered through respondent observations, and are reported numbers based on participant observations.

We analyzed different mitigation measures contributing to elephant casualties. There were eight observations of elephant injuries, and these were mainly due to trenches (12.8%). There were forty-seven observed elephant deaths, which were due to electric fences (38.3%) in Kerala, and solar fences (25.5%) in Karnataka (Fig. 3).

### Perspectives and emotions on seeing injured and deceased elephants

We found two emotions associated with experiences of seeing injured or deceased elephants: sadness and empathy. Results reported in Tables 2 and 3 and Supplementary Table 2 are based on the experiences and detailed accounts shared by the community.

**Table 2:** Most commonly expressed emotions on seeing injured and deceased elephants (*n* = 187 respondents).

Themes	Description	Exemplary quotes
Sadness (*n* = 87, 46.5% )	Feeling sad and upset upon seeing injured/deceased elephants	*“My eyes watered. It was a big creature. It is harming us*,* yes. But that is a different thing. It is a thing with a lot of prosperity. You have to take Mahalakshmi (hindu deity) on an elephant for prosperity”. (Participant 436)*
Empathy (*n* = 36, 19.3%)	Understanding the experiences and challenges of elephants navigating the landscape.	*“I feel sad for losing an animal. I feel the same when I lose any of my family members” (Participant 218)*
Pity for elephants (*n* = 28, 15.0%)	Pity towards elephants	*“We feel pity. If elephants are around we fear. When they die*,* we feel sad. It is also an animal. It was living somewhere. So*,* when it dies*,* we feel pity. Sometimes we feel it is fine. Or else it would attack us”. (Participant 215)*

**Table 3:** Major themes regarding respondent perspectives (*n* = 187 respondents) on seeing injured and deceased elephants.

Themes	Description	Exemplary quotes
Neutral (*n* = 23, 12.3%)	No particular perspective regarding elephants.	*“I didn’t feel anything. When city people see it*,* they may feel something*,* but I don’t because I keep seeing it all the time” (Participant 274)*
Elephants can be a nuisance (*n* = 7, 3.7%)	Elephants causing damage and being difficult to live in proximity to.	*“I feel if it dies*,* let it die. When it is alive*,* it comes and damages the crops and all*,* then we only curse it to die. If it is alive*,* it will damage our cropland only”(Particpant 105)*
Religious affiliation to the elephant God (*n* = 6, 3.2%)	Elephants are culturally and religiously significant to people based on ‘Ganesha’ (elephant-headed god in hinduism)	*“I feel pity*,* That is a poor animal. The God (Lord Ganesha) is killed." (Participant 194)*

The remaining (*n* = 313, 61.7%) responses did not comment on their perspectives due to never having seen an injured or deceased elephant.

### Recommendations on mitigation measures

There were 406 respondents (80.1%) that recommended interventions by authorities (i.e., Forest Department, Panchayat - Village Heads, State Government) (Table 4).

**Table 4:** Main mitigation measures and recommendations regarding authority interventions.the main mitigation measures (*n* > 10) have been mentioned based on frequency of occurrence across the 406 respondents. (Observations: 406 respondents, frequency mentioned: 470 total occurrences)

Mitigation measure	Description	Exemplary quotes
Railway line fencing (*n* = 105, 22.3%)	Installation of multiple railway tracks as a fencing measure to act as a barrier	*“It has been done in few places*,* they have placed railway pillars and fencing. If that is done*,* we can prevent elephants”. (Participant 202)*
Solar fencing (*n* = 95, 20.2%)	Fence powered by solar energy which can create a shock within the fence	*“The main problem is with elephants. For that*,* the forest boundaries should have flawless solar fencing. It should be done very well*,* in a way that’s useful for the public*,* it should be done. It shouldn’t be done partially*,* and then left incomplete. The work should be completed. It should include the locals too”. (Participant 354)*
Fencing (*n* = 88, 18.7%)	Creating fences (using wire and/or stone) to form a barrier	*“In the forest. It would be good if they can install a protective fence for it too”. (Participant 362)*
Trenches (*n* = 68, 14.5%)	A deep, narrow ditch that can create a depression in the ground	*“In my judgement*,* with EPT trench (elephant-proof trench)*,* railway fencing*,* and barricades with railway barricades*,* there should be solar also. If they do these three*,* then we can control them”. (Participant 031)*
Electric fencing (*n* = 32, 6.8%)	Fence powered by batteries which can create a shock within the fence	*“Electric fences along the paths we regularly use would be helpful and make us feel a little safe while we’re on our way to work. So that way*,* we can be sure that the elephants won’t block our way”. (Participant 406)*
Night guarding/patrolling (*n* = 17, 3.6%)	Authorities (i.e. Forest Departments) can help with patrolling farm lands at night, when the risk of crop damage is high	*“They have put a fence*,* it is deep*,* it comes in over that. They escape and come. Now*,* there are watchers*,* guards. They come and check here*,* the elephants come to the land and graze. They also come to guard”. (Participant 080)*

## Discussion

Our results underscore the complex interplay of socio-demographic and environmental drivers that influence how communities in southern India respond to human–elephant conflict, revealing both the determinants of mitigation deployment and the unintended consequences these measures may have on elephant populations. Unlike much of the existing literature that primarily focuses on conflict damages, this study broadens the scope by (a) examining the decision paths of implementing mitigation measures and (b) understanding how different mitigation techniques impact elephants themselves across diverse landscapes. As mitigation efforts gain traction globally^25,50^, our findings offer guidance for designing context-specific policies and management measures that balance human well-being with elephant conservation.

The results of the decision tree indicate that rainfall, acreage ownership and proximity to water bodies primarily influence the adoption of mitigation measures for elephant conflict, as shown in previous research^9,19,81^. Our findings indicate that Path (1), indicating moderate rainfall and land ownership, can influence people’s decision to deploy mitigation measures (68% likelihood). Seasonality and rainfall levels are critical environmental determinants, as elephants are highly sensitive to seasonal changes, and adjust their movement and foraging patterns in response to environmental cues^9,81^. These behavioral responses, coupled with climate variability and land-use transformations, bring elephants in proximity with agricultural areas, resulting in communities deploying mitigation measures to avoid damages^81^.

Moderate acreage ownership also increases the likelihood of mitigation measure deployment. This aligns with earlier studies showing that residents with more acres are more likely to invest in mitigation measures, such as building elevated huts on trees to monitor farms from a safe vantage point^82^. Many landowners use their fields for commercial agriculture, cultivating labor-intensive crops to sell in the market, and therefore deploy mitigation measures such as alarm deterrents and bee-box repellants^82,83^. Path (2) highlights both rainfall and acreage ownership as key determinants, along with distance to water body as a smaller node that influences mitigation measure deployment (7% likelihood). Prior research suggests that elephant movement patterns are mainly driven by water and food availability, often resulting in elephant movement in human populated spaces that are in proximity to water sources^18–20^. Research by Guarnieri et al., 2024 highlights that human–elephant conflict risks are likely to intensify for Asian elephants and African savanna elephants, within their current range boundaries, due to climate shifts, increasing human population densities and cropping patterns. These threats could escalate the pressures already faced by elephant populations, resulting in future conflict risks that threaten the species^81^.

The CART analysis results were consistent with our earlier findings in the literature regarding environmental drivers influencing mitigation decisions^26^. While we anticipated that socio-demographic characteristics would have a stronger impact, the contribution is relatively minor. Interestingly, there is some variable importance of the state-level effect (7.4%) between Karnataka and Kerala, suggesting limited variation in decision to implement mitigation between States, despite differing regional contexts such as administrative systems and socio-economic conditions. These results highlight the extent to which environmental variables are influencing conflict dynamics, which could be a reflection of the environmental pressures faced by elephants, and how this shapes decisions to adopt mitigation measures. The minor contribution of socio-demographic drivers and crop types, suggests that mitigation responses are more strongly driven by perceived environmental risks, such as seasonality changes, and resource limitations for elephants, particularly in conflict-prone landscapes^8,19,20^.

We also looked at reported mitigation measures that led to elephant injuries and deaths. For elephant injuries in Karnataka, trenches were most commonly reported, for elephant deaths, solar fences, and electric fences were referenced the most in Karnataka and Kerala, respectively. Research in Asia and Africa has highlighted that trenches can be dangerous for elephants, as they may sustain injuries or even die while attempting to climb out of deep trenches in which they become trapped^84,85^. Previous studies in Sri Lanka, and India have documented frequent cases of elephant electrocution by electric fences^82^. However, to our knowledge, no studies have examined elephant casualties of mitigation measures (i.e., trenches) within densely human-populated landscapes such as India.

Our findings also highlight multiple perspectives and emotions on seeing injured and deceased elephants, such as feelings of empathy, sympathy, and ambivalence. Some emotions described were socio-cultural being affiliated with religion, specifically the Hindu belief in the elephant god, Ganesha. Considering these communities frequently encounter elephants, and have experienced conflict events in the past, the persistence of these types of emotions and beliefs may provide insight into future management approaches. Previous studies have found links between religion and tolerance, such as research conducted by Kshettry et al., 2021, which identified that communities in West Bengal, India, were hesitant to apply for compensation after incurring damages due to reverence of the elephant god, ‘Mahakal’^86^. Similarly, a study in Bhutan highlighted that people who held Buddhist beliefs, and a ‘sacred’ value orientation were more tolerant to wildlife, especially to certain species that embodied a greater cultural significance (i.e., elephants and tigers)^87^. These insights also convey the complexity of human–wildlife interactions, and shed light on the nuances of these relationships. Coupled with socio-cultural beliefs, emotions are a cornerstone to comprehension of human–wildlife relationships, as emotions are fundamental for humans to cultivate everyday experiences^88,89^. Emotions can also influence decision-making processes and can ultimately shape an individual’s positionality on the spectrum of human–wildlife interactions^89^. In India, research conducted in Kerala identified that a forest-dwelling Adivasi community living in Wayanad Wildlife Sanctuary, were deeply tolerant and accepting of wildlife, indicating a deep coexistence due to perceiving wildlife as gods, teachers, relatives, and ultimately, equals in the forest^90^. Further, a study in Odisha located in India, highlights the different perspectives people have of wildlife, with children expressing a friendship with the wild elephants in the landscape, even being concerned for their wellbeing and safety^91^. By understanding community perspectives and emotions, we can establish baseline information on the intricacies of human–elephant relationships and the level of tolerance people have toward elephants in the landscape.

We also found mitigation measures and recommendations that local community members suggest regarding authority interventions, and identified that railway line fencing was commonly referenced as a suggestion followed by solar fencing, fencing (wire/stone), trenches, electric fencing and night patrolling. Railway line fencing has been observed as a useful barrier to prevent landscape accessibility for elephants in the past^12^, however, our results indicate that railway fencing has been associated with elephant injury (1.8% in Karnataka) and death cases (10.6% in Kerala) across both States. Previous research has also found that railway-line and electric fencing can often be ineffective in the long-term and even result in aggravating the situation^47^. With no previous research examining the impact of mitigation measures on wildlife injury and mortality in India, we conclude that trenches and electric fences are negatively impacting elephant populations by causing death and serious injury to wild elephants in the region.

Through our research, we have identified that people mainly reference barrier-based measures that prevent accessibility of elephants in the landscape. However, these measures can put an already threatened species at greater risk. Our findings reaffirm that no single mitigation measure is universally effective across all landscapes and elephant conflict contexts. The effectiveness of mitigation measures is highly dependent on environmental, socio-economic and governance factors^15,25,82^. In the study area, mitigation measures such as solar, electric and wire fencing were implemented and were relatively effective in reducing crop damages, however, despite the low cost and maintenance, these measures can occasionally be poorly installed and harmful to elephants, as evidenced by our results. Although firecrackers are used as an active deterrent in conflict situations, our findings suggest that firecrackers can contribute to elephant deaths. Previous research has shown that these explosives can cause fatal injuries to elephants during conflict encounters, resulting in elephants being wounded and succumbing to death^54^. Previous research has also indicated that people chasing elephants using firecrackers can cause susceptibility to injury or death of the person, escalating cycles of hostility or even retaliation^30^.

Instead, we propose that conservation measures focus on non-lethal, preventative, proactive measures, such as early warning systems (information about elephant movement conveyed through local television channels and broadcast SMS sent directly to local communities) that have the capacity to inform community members about elephant movement in real-time, while also avoiding serious conflict casualties to people and elephants. Kumar & Raghunathan (2014), found that 36 of 41 cases of human death events occurred due to a lack of early warning systems in Valparai, Tamil Nadu, in India^92^. In addition to early warning systems, as suggested by community members, the implementation of night patrolling by management could be beneficial in preventing elephants from entering farmlands, as strobe lights, speakerphones, and other similar measures, have been effective in certain cases during patrolling, as observed with elephants in Botswana^93^. Further, technological advancements such as artificial intelligence (AI) integrated with thermal infrared cameras could be utilized for biodiversity monitoring in densely populated areas. Emerging research globally highlights the potential of AI and thermal imaging technologies in enhancing species monitoring, deterring poaching, and supporting long-term habitat and wildlife assessments^94,95^. When applied effectively, these technologies can function as robust early warning systems and strengthen ongoing conservation monitoring efforts^94,95^. In addition to technological and environmental measures, financial mechanisms, such as *ex-gratia* compensation schemes offered by governments can help offset the economic losses incurred to individuals affected by human–elephant conflict^47,86^.

Conflict management measures should also incorporate outreach workshops and training programs that focus on disseminating knowledge about environmental solutions, such as regenerative farming, sustainable agriculture and agroforestry practices. These approaches can help reduce human–elephant conflict, by promoting soil and water conservation, enriching habitat connectivity for elephants, and mitigating certain impacts of climate change, such as extreme rainfall variability. Previous research on human–elephant conflict has emphasized the value of these solutions, advocating for polycropping and integrated agroforestry models to restore degraded land and increase the economic viability of farms^96^. Mukomberanwa et al., 2025, identified key areas of regenerable land in semi-arid savannah ecosystems that could support the African savannah elephants migratory and foraging requirements. Their findings suggest that combining habitat connectivity modeling with land regeneration efforts can enhance fodder availability within protected areas, reducing landscape fragmentation and alleviating conflict^97^. Consequently, further research is needed to assess the potential for habitat restoration in fragmented landscapes of the Western Ghats. Given that elephant habitats in Karnataka are contiguous with forests in Kerala and Tamil Nadu, this region holds significant conservation value and offers opportunities to strengthen habitat corridors across state boundaries.

The United Nations Population Division has projected that the human population will grow to 9.77 billion by 2050^98^. Consequently, human–elephant conflict is likely to intensify, especially in biodiversity hotspots like the Western Ghats, where people and elephants increasingly share space^35^. The challenges of conflict will continue to fall on both, local communities and the elephants navigating these fragmented landscapes. Addressing this challenge requires a nuanced understanding of the drivers behind conflict, along with community perspectives on where and how mitigation measures should be implemented. By integrating technological innovations with environmental insights, we can move toward proactive, context-specific measures that balance the needs of people with the long-term survival of elephants in shared landscapes.

## Caveats and future research

Our study skewed towards men compared to women, due to women being hesitant to answer questions^99^. Women would often request the man in the household to participate in the questionnaire. Our team always consisted of at least one man (our team size was 2–3 people) which could have possibly made women feel uncomfortable and unwilling to speak. Future studies could focus on building community relationships by establishing team members in the landscape for longer periods of time to build communication and transparency. Our study highlights the key drivers across socio-demographic and environmental aspects, however, future research can be done to identify other interdisciplinary drivers (i.e., socio-cultural) which could be a component factored into future management plans. Future research can also delve into the efficacy of mitigation techniques, which could aid conservation practitioners and NGOs focused on conserving elephants and minimizing the adverse impacts to local communities in diverse landscapes.

## Supplementary Information

Below is the link to the electronic supplementary material.

Supplementary Material 1

Supplementary Material 2

Supplementary Material 3

Supplementary Material 4

## Data Availability

The raw data utilized in this article will be made available by the corresponding author (simranprasaduae@gmail.com) upon reasonable request.
