# Supplementary material for: Community mitigation decisions in elephant conflict zones of southern India depend on environmental and socio-economic drivers
Source: Sci Rep. 2025 Oct 6;15:34693. doi: 10.1038/s41598-025-14867-3 (PMC12500949; doi:10.1038/s41598-025-14867-3)
Supplement: Supplementary file 4 — Supplementary Material 4 [file 41598_2025_14867_MOESM4_ESM.docx]

**Supplementary Section**

**
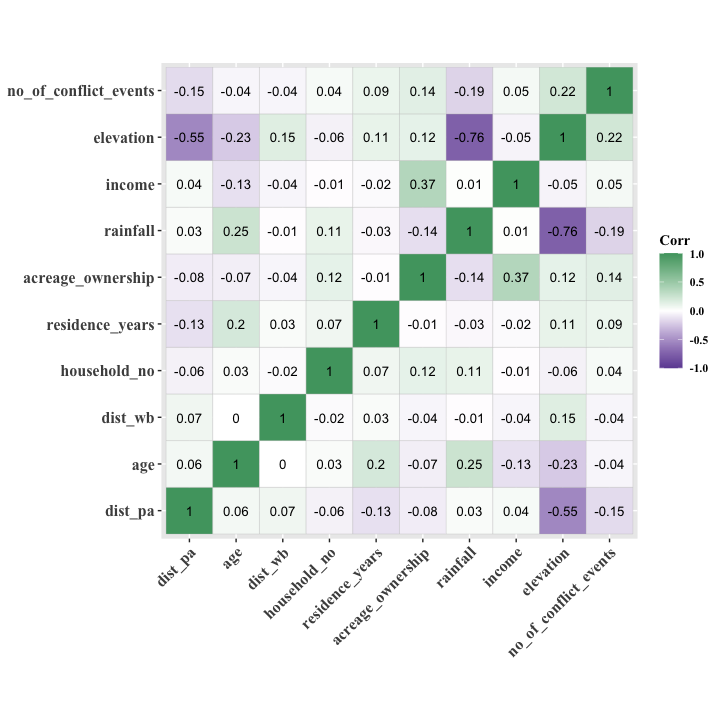
**

**Supplementary Fig 1:** A correlation plot of all the numerical covariates used to model socio-demographic and environmental drivers that influence the ability to deploy, or not deploy mitigation measures. The color gradients used in Supplementary Fig 1 indicate the strength of association between the different covariates used in the modeling. Values ranged from -0.75 to 0.37.


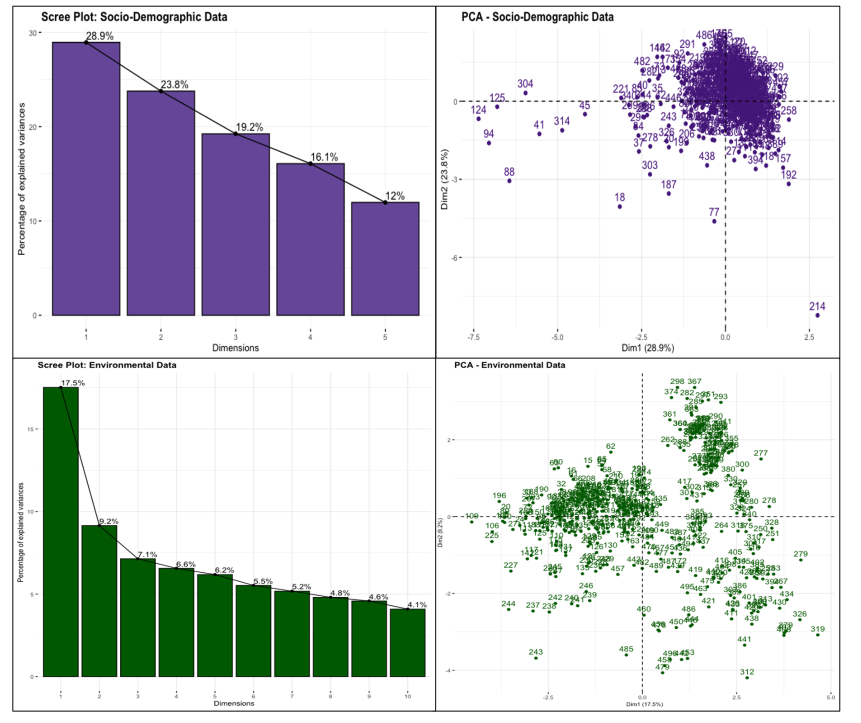


**Supplementary Fig 2:** Grid of scree plots and visualizations illustrating the variance of data.

**Supplementary Table 1 :** Participant characteristics

| **Categories** | **Sub-categories** | **Number** | **Percentage** |
| --- | --- | --- | --- |
| Age | 21 - 35 | 89 | 18.9% |
|  | 36 - 50 | 152 | 32.3% |
|  | 51 - 65 | 157 | 33.4% |
|  | >65 | 72 | 15.3% |
| Gender | Men | 115 | 24% |
|  | Women | 355 | 76% |
| Education | Uneducated | 79 | 16.8% |
|  | Primary school | 201 | 42.8% |
|  | Secondary school | 145 | 30.9% |
|  | Bachelor Degree | 39 | 8.3% |
|  | Master’s Degree | 6 | 1.3% |
| Various income sources | Agriculture | 347 | 73.8% |
|  | Wage labor | 55 | 11.7% |
|  | Other sources (grocery clerks, fishermen, etc.) | 27 | 5.7% |
|  | Salaried job | 11 | 2.3% |
|  | Dairy farming | 10 | 2.1% |
|  | Rubber tapping | 8 | 1.7% |
|  | Pension | 6 | 1.3% |
|  | No income | 4 | 0.85% |
|  | Wage labor and dairy farming | 2 | 0.42% |
| Household size | 1 - 5 | 342 | 72.8% |
|  | 6 - 10 | 120 | 25.5% |
|  | >10 | 8 | 1.7% |
| Number of residence years near the forest | 1 - 10 | 29 | 6.2% |
|  | 11 - 40 | 198 | 42.1% |
|  | 41 - 60 | 165 | 35.1% |
|  | >60 | 78 | 16.6% |
| Mitigation Measures deployed | Yes (Karnataka) | 227/253 | 89.7% |
|  | Yes (Kerala) | 126/254 | 49.6% |
| Acreage ownership | 0 - 5 | 396 | 84.2% |
|  | 6 - 10 | 54 | 11.5% |
|  | 11 - 25 | 15 | 3.2% |
|  | >25 | 5 | 1.1% |
| Average annual yearly income (INR)  83.95 INR = 1 USD (conversion rate as of October 2024). | 0 - 10,000 (USD $0 - $199) | 25 | 5.3% |
|  | 10,001 - 30,000 (USD $119.1 - $357) | 64 | 13.6% |
|  | 30,001 - 50,000 (USD $357.4 - $595) | 76 | 16.2% |
|  | 50,001 - 100,000 (USD $595.6 - $1,191) | 102 | 21.7% |
|  | 100,000 - 500,000 (USD $1,191.2 - $5,955) | 182 | 38.7% |
|  | >500,000 (>USD $5,955.1) | 21 | 4.5% |

**Supplementary Table 2:** More quotes for each theme regarding perspectives on seeing injured and deceased elephants (n = 187 respondents).

| **Themes** | **Theme descriptions** | **Exemplary Quotes** |
| --- | --- | --- |
| Sadness (n = 87, 46.5% ) | Descriptions of feeling sad and upset upon seeing injured/deceased elephants | *“I feel very sad. They don’t understand the dangers of coming here and sometimes, they get injured or die because of it.” (Participant 420)* |
| Empathy (n = 36, 19.3%) | People describe understanding the experiences and challenges of elephants navigating the landscape. | *“The humane feeling anyone would feel. It’s something that died, right? There is no celebration or happiness. They are a life form too, right?”. (Participant 499)* |
| Pity for elephants (n = 28, 15.0%) | Descriptions of pity towards elephants | *“Even if it causes harm, it is still a poor creature that lost its life”.(Participant 490)* |
| Neutral (n = 23, 12.3%) | No particular emotion or perspective regarding elephants. | *“I don’t feel anything in particular”. (Participant 498)* |
| Elephants can be a nuisance (n = 7, 3.7%) | Descriptions of elephants causing damage and being difficult to live in proximity to. | *“I feel like some nuisance has gone away now”. (Participant 500).* |
| Religious affiliation to the elephant God  (n = 6, 3.2%) | People believing elephants are culturally and religiously significant to them based on ‘Ganesha’ (elephant-headed god in Hinduism) | *“It feels so sad. We are all the devotees of Lord Ganesha. But when elephants make trouble for us, we will feel hate towards them. I love them, but also have fear ". (Participant 433)* |
